# From molecular design to clinical translation: dual-targeted CAR-T strategies in cancer immunotherapy

**DOI:** 10.7150/ijbs.108036

**Published:** 2025-03-31

**Authors:** Zhenrong Wang, Mengyi Wang, Mengting Wang, Ruijie Zhou, Xiaotong Deng, Xin Ouyang, Minghui Chu, Xinyu Wei, Lei Yang, Jinbiao Liu, Yao Xu

**Affiliations:** 1 Institute of Biology and Medicine, College of Life Science and Health, Wuhan University of Science and Technology, Wuhan, Hubei 430081, China.; 2 National “111” Center for Cellular Regulation and Molecular Pharmaceutics, Key Laboratory of Fermentation Engineering (Ministry of Education), Hubei Provincial Cooperative Innovation Center of Industrial Fermentation, Hubei Key Laboratory of Industrial Microbiology, Sino-German Biomedical Center, Hubei University of Technology, Wuhan, Hubei, 430068, China.; 3 Institute of Medical Microbiology, Jinan University, Guangzhou 510632, China.; 4 People's Hospital of Jingyang County, Xianyang, Shaanxi, 713700, China.

**Keywords:** dual target, CAR-T cell therapy, leukemia, solid tumor, immunotherapy

## Abstract

The pathogenesis of tumors involves various abnormalities at both the cellular and genetic levels. Chimeric antigen receptor (CAR)-T cell immunotherapy has emerged as a transformative treatment strategy that effectively addresses these challenges. While CAR-T therapy has shown remarkable success in treating hematological malignancies, limitations have been identified, particularly in single antigen-targeting CAR-T therapies. These limitations include antigenic mutation or loss, reduced efficacy against leukemia, and poor results in solid tumors due to factors like low CAR-T cell persistence, limited tumor infiltration, rapid cell exhaustion, the suppressive tumor microenvironment, and heterogeneous tumor antigen expression. In recent years, multi-antigen targeted CAR-T therapies have garnered significant attention for their potential to prevent tumor relapse and progression. This review outlines the fundamental design of dual CAR structures and summarizes the major advancements in both preclinical studies and clinical trials of dual-targeted CAR-T cell therapy, categorized by cancer type. Additionally, it discusses the challenges associated with dual-targeted CAR-T therapy and the strategies to enhance its efficacy and applicability in treating both hematologic and solid tumors. In conclusion, the progress in dual-targeted CAR-T cell therapy presents a promising therapeutic avenue for multiple malignancies, offering insights into future modifications of immunotherapy to advance the field.

## Introduction

Cancer remains one of the most pressing global health challenges, with an estimated 20 million new cases and 9.7 million deaths reported in 2022 1, primarily due to its high metastasis rate and poor prognosis. Despite significant advancements in medical technology and clinical strategies, including surgery, radiotherapy, and chemotherapy, several limitations still impact therapeutic outcomes. These limitations include severe side effects, high recurrence rates, and the development of drug resistance 2, 3. In recent years, cell immunotherapy, which harnesses and enhances the immune system to target and eliminate cancer cells, has emerged as a promising cancer treatment approach, demonstrating immense potential and unique advantages for reducing relapse rate 4.

Chimeric antigen receptor T (CAR-T) cell therapy has revolutionized cancer treatment, particularly for hematological malignancies. With over 80% complete remission rates in relapsed/refractory B-cell acute lymphoblastic leukemia (B-ALL) and 40-50% long-term survival rates in diffuse large B-cell lymphoma (DLBCL), CAR-T therapy has demonstrated unprecedented success where conventional therapies have failed 5, 6. This groundbreaking approach leverages genetically engineered T cells to precisely target and eliminate cancer cells, offering a paradigm shift in immunotherapy. However, the clinical application of single-antigen targeted CAR-T cells faces significant challenges, including antigen escape, therapy-related toxicities (e.g., cytokine release syndrome), and limited efficacy in solid tumors 7, 8.

Given these limitations, recent evidence has proposed dual-CAR-T cell therapies, which simultaneously express two distinct CAR structures on a single T cell, enabling the modified CAR-T cells to recognize two different antigens on tumor cells 9, 10. Compared to single-target CAR-T therapy, the dual-targeting strategy can enhance the specificity and effectiveness of cancer therapy by reducing the risk of antigen escape and promoting immune cell infiltration 11. This article reviews the structural properties of dual-targeted CAR-T cells and highlights the potential advantages of dual CAR over single CAR structure. Moreover, we discuss recent innovations in preclinical studies and clinical trials for both hematological malignancies and solid tumors, as well as the challenges and future prospects for enhancing the efficacy and applicability of dual-targeted CAR-T cell therapy in treating cancer.

This review aims to provide a comprehensive overview of dual-targeted CAR-T cell therapies. The review begins by describing three main types of dual-targeted CAR-T structures. We then summarize the research progress of dual-targeted CAR-T therapies in hematologic malignancies and solid tumors, respectively. Finally, we address the challenges associated with the application of dual-targeted CAR-T cells and propose potential strategies, while also discussing the future prospects for their broader clinical translation.

### Overview of CAR-T therapeutic issues

CAR-T cell therapy has shown significant therapeutic effects in treating B-cell malignancies. However, in some patients, tumor cells can evade CAR-T cell attacks through multiple mechanisms, including antigenic modulation, epitope masking, and lineage switching. For instance, in pediatric B-cell acute lymphoblastic leukemia (B-ALL), CD19-negative relapse occurs in approximately 10-30% of patients post-CAR-T therapy due to CD19 downregulation or loss, often mediated by alternative splicing of CD19 mRNA or clonal selection of pre-existing CD19-negative subpopulations 12. The mutation or loss of tumor antigens is a prevalent mechanism of resistance to single-target CAR-T cell therapy in B-cell malignancies. In solid tumors, the limitations of single-target CAR-T therapy are even more pronounced due to the high heterogeneity of tumor antigens and the complex tumor microenvironments (TME), which is composed of a range of immunosuppressive factors, including regulatory T cells, myeloid-derived suppressor cells, and tumor-associated macrophages, all of which can dampen CAR-T cell function 13. Overall, the challenges of single-target CAR-T cell therapy are multifaceted and can be attributed to various factors, including the antigen escape and tumor heterogeneity, immunosuppressive TME, limited CAR-T cell persistence and exhaustion, and therapy-related toxicities 8, 14-17. These limitations underscore the need for multi-antigen targeting strategies, such as dual-targeted CAR-T cells, to overcome antigen escape and enhance therapeutic efficacy.

### Advantages of dual-targeted CAR-T therapy relative to single CAR

Dual-targeted CAR-T cell therapy is an innovative approach in cancer treatment that involves engineering CAR-T cells to express two distinct antigen recognition domains, enabling them to target two different antigens on tumor cells simultaneously. This therapeutic strategy offers several advantages over traditional single-target CAR-T cell therapy 9, 18-21. (1) Reduced potential for antigen escape: By targeting two antigens concurrently, dual-targeted CAR-T cell therapy reduces the likelihood of tumor cells evading immune attack through the loss or downregulation of a single antigen. (2) Enhanced therapeutic efficacy: Dual-targeted CAR-T cells can recognize and attack tumor cells via two distinct mechanisms, thereby amplifying the overall antitumor activity and potentially leading to more effective treatment outcomes. (3) Improved treatment specificity: The specificity of clinical therapy is significantly increased by targeting two antigens, which helps minimize damage to normal cells and may reduce the side effects commonly associated with traditional cancer treatments. (4) Potential synergistic effects: The combination of two CAR structures can generate synergistic interactions, enhancing cytotoxicity and improving the efficiency of tumor cell elimination. (5) Broader application range: Dual-targeted CAR-T cell therapy may be applicable to a wider range of tumor types, particularly those with insufficient single antigen expression or exhibiting antigen heterogeneity.

## Dual CAR structures

**Figure 1:**
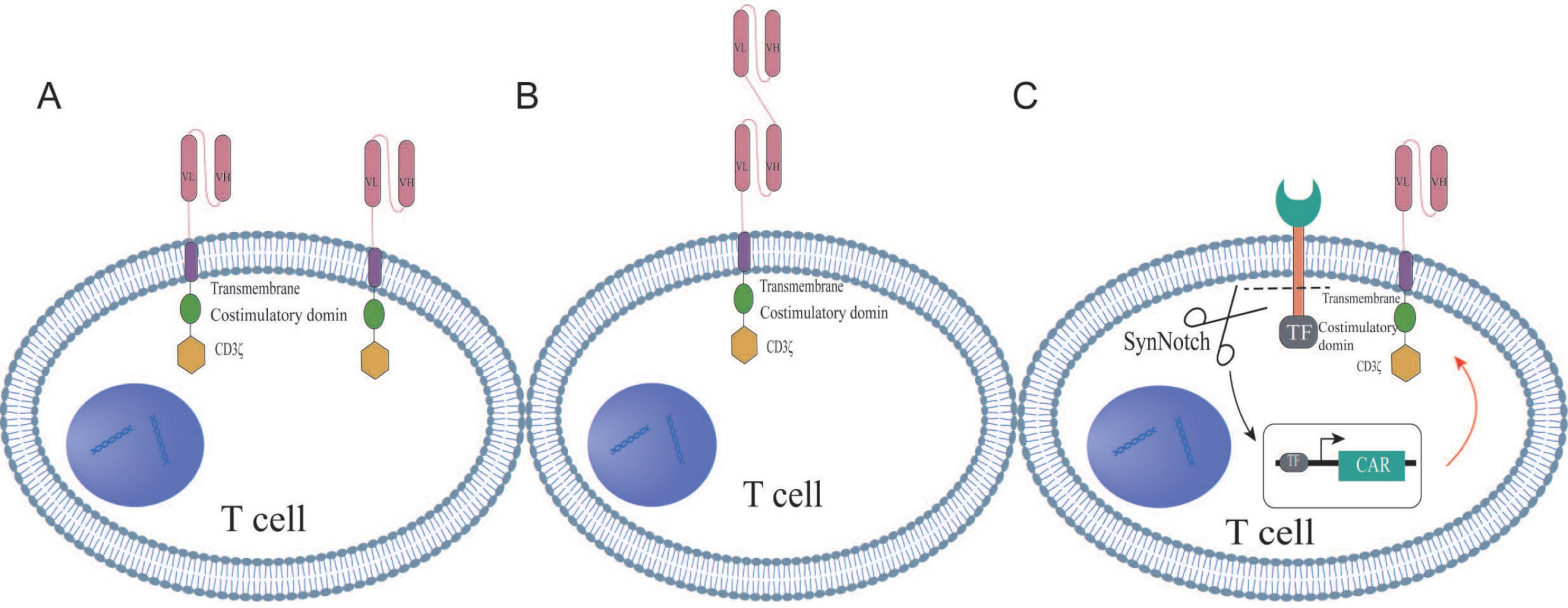
The schematic structures of dual-targeted CAR-T cells. (A) Tandem dual CAR structure. (B) Parallel dual CAR structure. (C) synNotch dual CAR structure.

Dual-targeted CAR-T cell therapy, which introduces two distinct chimeric antigen receptors (CARs) on a single T cell, has emerged as an innovative strategy for cancer treatment. These bispecific CAR constructs are designed to enable T cells to simultaneously recognize and target two different antigens on tumor cells, thereby enhancing both the specificity and potency of the immune response. The structural design of dual CAR-T cells can be primarily categorized into three types: tandem, parallel, and synNotch configurations. Tandem CAR-T cells feature a single expression unit containing two CAR domains in tandem, allowing T cells to activate a cascade of amplification signals in response to the second antigen after the initial antigen recognition of the first antigen (Figure 1A). In contrast, parallel CAR-T cells express two independent CAR structures on the same T cell, permitting simultaneous recognition and targeting of two distinct antigens (Figure 1B). The synNotch structure mimics the signaling mechanism of the natural T cell receptor, enabling selective signal activation in dual CAR-T cells upon recognition of a specific antigen (Figure 1C). These diverse structural designs reflect the ongoing exploration and innovation by researchers to enhance the safety of dual CAR-T cell therapy, reduce toxicity, and improve therapeutic efficacy. The differences among the three CAR structures, such as target recognition, signaling pathways, and potential advantages, were summarized in Table S1. In the following sections, we will thoroughly explore the characteristics, advantages, and clinical potential of the three configurations.

### Dual CAR tandem structures

The second-generation CAR structure is modified to construct the tandem dual CAR molecule, which consists of two distinct antigen recognition domains targeting different epitopes, along with a spacer sequence, a transmembrane domain, a co-stimulatory domain, and a signaling domain. Specifically, the antigen recognition domains are typically single-chain variable fragments (scFvs); the spacer sequence connects the dual scFvs to the co-stimulatory and signaling domains, often utilizing the hinge region of CD8α; the transmembrane domains are primarily derived from CD8α and CD28; while the co-stimulatory domains commonly include CD28 and 4-1BB; CD3ζ serves as the signaling domain. Most dual-targeting scFvs are linked by a unique connector, with the glycine-serine linker (Gly4Ser) being the most widely used 22, 23. This linker, composed of repetitive glycine and serine residues, provides a flexible bridge that reduces steric hindrance, allowing the connected proteins to fold and function independently. This flexibility enables the heavy and light chains, linked by the Gly4Ser linker, to form a complete antibody structure, optimizing antigen recognition and binding. Previous studies have also incorporated a reporter gene after CD3ζ through a T2A cleavage site to assess the transduction efficiency of the engineered cells. For example, Schmidts et al. 20 linked a mCherry reporter gene, while Dai et al. 22 connected EGFRt to the CAR fragment via a T2A sequence.

### Dual CAR parallel structures

The parallel dual CAR molecular structure utilizes a second-generation design, featuring two distinct receptor targets that are independently expressed on T cells. This structure sequentially comprises the antigen recognition domains for two different targets, a hinge region, a transmembrane domain, co-stimulatory domains, and a signaling domain 24, 25. The antigen recognition domains are primarily composed of scFvs, while the hinge region connects the scFvs of the two distinct targets to the co-stimulatory and signaling domains, typically utilizing the hinge region of CD8α to optimize spatial flexibility and antigen-binding efficiency 26. The transmembrane domains are predominantly derived from CD8α and CD8, with CD28 also serving as a transmembrane domain. The common co-stimulatory domains include CD28 and 4-1BB4, and CD3ζ functions as the signaling domain. In parallel CAR design, CD28 and 4-1BB are frequently used as co-stimulatory domains to activate T cells, playing a pivotal role in T cell activation and proliferation. Each co-stimulatory unit is independently expressed and strategically positioned near the T cell plasma membrane, thereby mimicking the natural arrangement of T cell receptors (TCRs) and co-stimulatory receptors under physiological conditions 27. Previous studies have reported that other co-stimulatory domains, such as CD137 and ICOS, can enhance CAR-T cell persistence and effector functions by coordinately activating metabolic pathways 28, 29. The hinge region can consist of either the CD8 hinge or the IgG4mt hinge, while the signaling domain is most commonly CD3ζ. In research involving parallel dual-targeted CAR-T cells, investigators have adjusted the order of scFvs and the signaling domains to enable a single T cell to simultaneously recognize and target two distinct tumor-associated antigens 30.

### Dual CAR synNotch structures

In the synNotch structure, the antigen-specific scFv is linked to the Notch core and transcription factor. In general, synNotch receptors contain an N-terminal CD8α signal peptide (MALPVTALLLPLALLLHAARP) for membrane targeting, as well as an α-myc tag (EQKLISEEDL) or flag tag (DYKDDDDK) or GFP tag or BFP tag for detecting surface expression 31. However, some synNotch receptors are engineered by attaching the humanized antigen scFv to the intracellular Notch core domain, which is fused with the tTA transcription factor 32. The intracellular domain of the synNotch structure may also include a fusion protein comprising a DNA-binding domain and transcription activator, with the upstream activation sequence (UAS) integrated into the receptor's structural elements. Additionally, markers such as blue fluorescent protein (BFP) are incorporated into T cells for efficient cell sorting 33.

In conclusion, the tandem structure of dual-targeted CAR-T cells positions two CAR domains in series within a single expression unit, allowing T cells to first recognize the initial antigen and then, through a cascade amplification mechanism, activate a response to the second antigen. This design enables T cells to simultaneously recognize and target two distinct tumor antigens, thereby enhancing their ability to identify and eliminate tumor cells 34. In contrast, the parallel structure of CAR-T cells expresses two independent CARs on the same T cell, enabling simultaneous recognition and attack of two different antigens. The design of parallel CAR-T cells is of significant importance, as it not only provides potent anti-tumor activity but also helps mitigate T cell exhaustion and senescence, thereby improving the longevity and functionality of T cells 35. The synNotch CAR represents an innovative CAR structure that mimics the signaling mechanism of natural Notch receptors, allowing precise control over T cell activity. It combines two distinct antigen-binding domains, where recognition of the first antigen triggers a Notch receptor-like cleavage and release, thereby activating the expression of the second CAR and facilitating the recognition and response to the second antigen 36. The synNotch CAR circuit, which targets highly specific solid tumor antigens, enhances both specificity and therapeutic efficacy by modulating T cell exhaustion. This approach not only improves specificity through multi-antigen sensing but also provides a universal strategy for enhancing efficacy through cell-autonomous and context-dependent regulation of CAR expression. A previous *in vivo* study revealed that the synNotch CAR significantly maintained T cell memory subset by preventing tonic signaling, which is crucial for the durability and sustained activity of cell therapies 37.

## Dual-targeted CAR-T cell therapy in oncology

**Figure 2:**
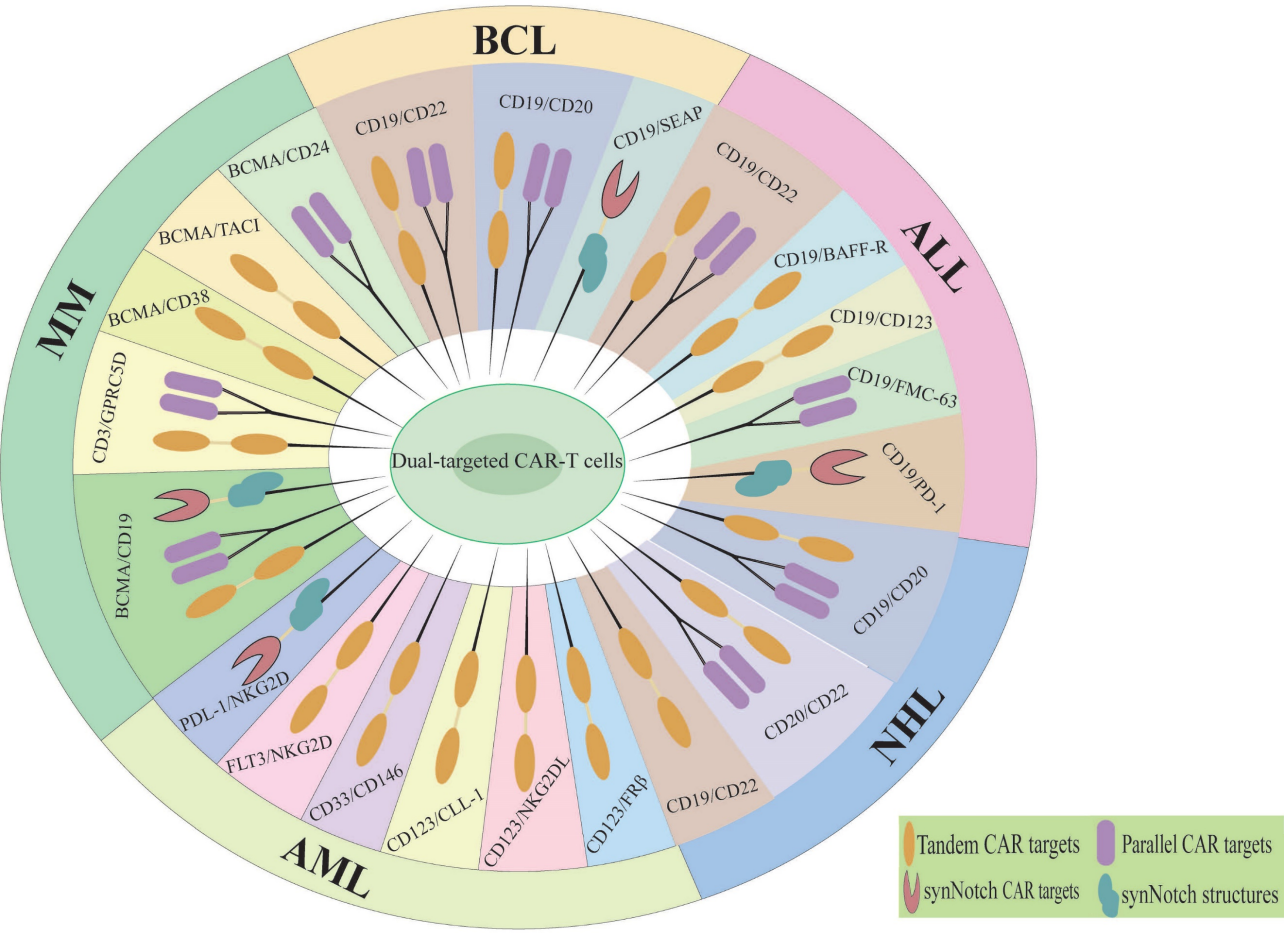
Dual therapeutic targets of CAR-T cell therapy in hematologic malignancies. MM, Multiple myeloma; BCL, B-cell lymphoma; ALL, Acute lymphoblastic leukemia; NHL, Non-hodgkin lymphoma; AML, Acute Myeloid Leukemia.

**Figure 3:**
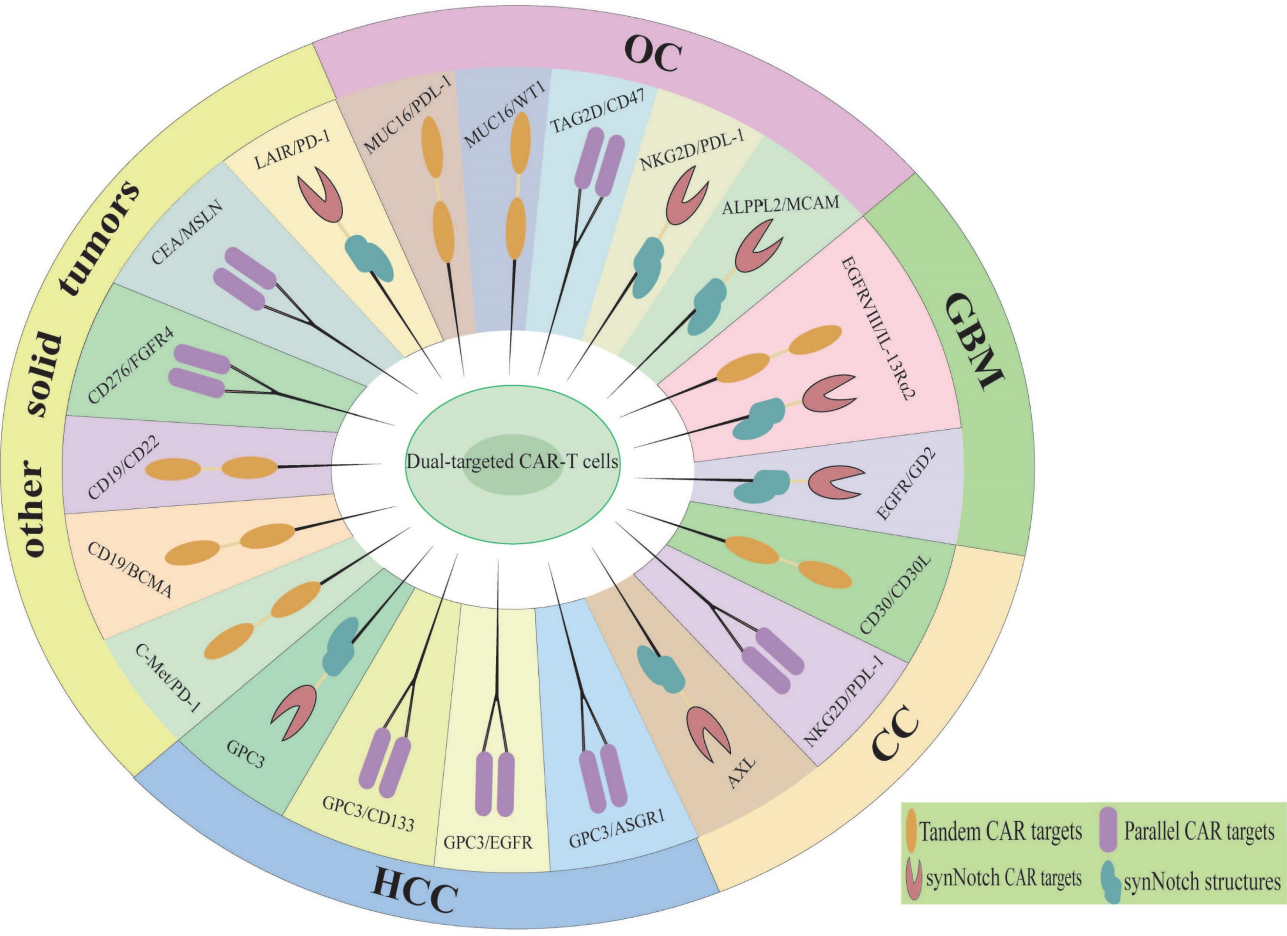
Dual therapeutic targets of CAR-T cell therapy in solid tumors. HCC, Hepatocellular carcinoma; CC, Cervical cancer; GBM, Glioblastoma multiforme; OC, Ovarian cancer.

Dual-targeted CAR-T cells represent a promising strategy to address the limitations of single-target therapies, particularly in overcoming resistance mechanisms such as antigen escape, TME suppression, and T cell exhaustion 38. By simultaneously targeting two tumor-associated antigens, dual-targeted CAR-T cells significantly reduce the risk of antigen loss or downregulation, a major cause of relapse in single-target therapies. In solid tumors, dual-targeting strategies that co-target tumor antigens and TME components have shown enhanced T cell infiltration and reduced immunosuppression 39. Additionally, the incorporation of optimized co-stimulatory domains (e.g., CD28 and 4-1BB) improves T cell persistence and metabolic fitness, mitigating exhaustion 11. The main diseases and dual targets for dual CAR-T cell therapy in hematological malignancies are illustrated in Figure 2 and Table 1. Although the application of dual CAR-T cell therapy remains in the early stages of exploration, studies have shown favorable safety profiles and preliminary antitumor activity across various solid tumor types (Figure 3, Table 2), including ovarian cancer and hepatocellular carcinoma. The results of these preclinical and clinical trials provide a strong foundation for expanding the use of dual CAR-T cell therapy to a broader range of cancers and open new avenues for future therapeutic strategies.

### Applications of Dual CAR in hematologic tumors

#### Multiple myeloma

Multiple Myeloma (MM), which accounts for approximately 10% of hematological malignancies, is characterized by abnormal proliferation of plasma cells in the bone marrow, often accompanied by end-organ damage such as acute kidney injury, anemia, destructive osseous bone lesions, and hypercalcemia 50. Despite the widespread use of novel therapeutic agents, including proteasome inhibitors, monoclonal antibodies and immunomodulatory factors, to improve survival outcomes, the relapse rate remains high, and prognosis is poor due to severe chemoresistance 51. Currently, two anti-BCMA CAR-T cell products have been approved by the US Food and Drug Administration (FDA) for the treatment of relapsed or refractory (R/R) MM. While BCMA-targeted CAR-T cell therapy has shown high initial response rates, its clinical efficacy is often limited by the temporary nature of responses and frequent relapses, with a median progression-free survival of only 12.2 months, underscoring challenges in achieving long-term curative outcomes due to factors such as CAR-T cell persistence, antigen escape, and the hostile tumor microenvironment 52. In light of these challenges, emerging preclinical and clinical strategies involving dual-targeted CAR-T cells are being explored to address the limitations of current MM treatment.

G-protein-coupled receptor family C group 5 member D (GPRC5D), a 7-pass transmembrane receptor protein encoded by the *GPRC5D* gene, has been identified as a potential target to prevent BCMA escape-mediated MM relapse. Single-cell whole-genome sequencing has helped uncover this target, and an FDA-approved antibody targeting GPRC5D (talquetamab) has shown promising response rates in a phase I clinical study, albeit with a distinctive side effect profile 53. This has led to the exploration of CAR-T designs targeting both BCMA and GPRC5D. Three structural approaches have been investigated; (1) pooled production of single-target CAR-T cells, (2) two distinct CARs from a single vector with bicistronic elements, and (3) the dual-scFv "single-stalk" CAR design. Among these, pooled single CAR-T and bicistronic CAR-T cells exhibited the highest efficacy against BCMA-negative disease. Notably, the bicistronic design proved more effective for diseases co-expressing both BCMA and GPRC5D, highlighting the enhanced therapeutic efficacy of dual-targeted CAR-T cells through intensified interaction with tumor cells 10. Another potential cause of relapse in MM could be the high expression of CD19 on residual plasma cells, which may drive myeloma propagation and chemotherapy resistance 54. A novel tandem bispecific CAR targeting both CD19 and BCMA has been shown to induce cytotoxic effects *in vitro* and tumor regression in xenograft model. This approach resulted in higher anti-tumor efficacy and reduced subsequent recurrence compared to conventional single scFv-CAR-T cells 55. Recently, an open-label, single-arm phase I/II clinical trial (ChiCTR2000033567) enrolled 50 MM patients, who were treated with BCMA/CD19 dual-targeted CAR-T cells. Of the 46 patients who achieved an overall response (92%), 27 patients with partial remission (PR) or better presented sustained responses, with a 1-year progression-free survival (PFS) rate of 55% during an 11-month follow-up 44. Additionally, considering that minimal residual myeloma cells express stem-like genes such as CD24, Sun et al. 56 constructed a bispecific BCMA-CD24-CAR-T cell therapy and revealed that this dual-targeted CAR-T cells exhibited increased cytolytic activity and prolonged survival in xenograft models compared to monospecific anti-BCMA CAR-T treatment. Furthermore, several combination targets for CAR-T therapy in MM are under investigation, building on the success of bispecific antibodies, including BCMA/CD3, GPRC5D/CD3 and BCMA/CD19 57.

#### B-cell malignant tumor

B-cell malignancies, primarily B-cell lymphomas (BCL), include B-cell Hodgkin's lymphoma and B-cell non-Hodgkin's lymphoma (B-NHL). The typical clinical manifestations of B-cell malignancies include nausea, vomiting, and headaches, often due to elevated intracranial pressure 58. Diffuse large B-cell lymphoma (DLBCL) is the most common subtype of B-NHL, with a median age at diagnosis of 66 years. In the United States and Western Europe, the number of new DLBCL cases is expected to increase from 29,108 in 2020 to 32,443 in 2025 59. Despite recent advancements in clinical treatments, 30% to 40% of patients ultimately succumb to severe complications. Currently, three CAR-T cell products—Axicabtagene ciloleucel (axi-cel), lisocabtagene maraleucel (liso-cel), and tisagenlecleucel (tisa-cel)—have been approved for R/R LBCL patients who have received three or more prior lines of therapy 60. While CAR-T therapy demonstrates curative potential, limited accessibility and stringent eligibility criteria restrict the number of patients who can benefit from this treatment.

The development of CD19 CAR-T cell therapy has provided significant treatment options for B-cell malignancies, improving survival rates and reducing side effects. However, the loss or mutation of CD19 antigen epitopes remains a major cause of disease relapse 61, 62. CD22, a member of the sialic acid-binding immunoglobulin-like lectin family, is highly expressed in most B-cell malignancies and has been identified as a potential target to synergize with CD19 in CAR-T cell therapy to prevent cancer recurrence 63. In this context, four different structures of CD19/CD22 dual-targeted CAR-T cells, with varying linkers and antibody sequences, were designed and compared. The bispecific CAR-T cells with an EAAAK linker exhibited superior pharmacological effects, including enhanced cytotoxicity and higher levels of cytokine secretion, compared to those with a G4S linker. Additionally, dual-targeting or sequential administration of CD19/22 CAR-T cell therapies has been explored to overcome relapses caused by CD19-negative tumor cells in B-NHL patients. However, most patients fail to achieve durable responses, partly due to CAR-T cell exhaustion driven by the PD-1/PD-L1 pathway. To address this issue, a prospective clinical trial combining dual-targeting CD19/22 CAR-T cells with the anti-PD-1 antibody (tislelizumab) was conducted for treating R/R B-NHL 64. The results indicated that this combination therapy induced safe and durable responses, significantly improving patient prognosis. Epcoritamab and Glofitamab, two bispecific antibodies for CD3 and CD20, were approved by the FDA in May and June of 2023, respectively, for the treatment of DLBCL. In a phase I/II clinical trial involving R/R NHL, 22 DLBCL patients received a full dose of Epcoritamab, with 15 (68%) achieving a positive response. Among these, 10 (45%) patients had a median follow-up of 9.3 months. The ORR was 75%, with a CR rate of 69%. Epcoritamab also induced remissions in patients with aggressive diseases refractory to first-line and/or last-line treatments, with no grade 3 toxicities reported, further supporting its safety profile 65. Similarly, Glofitamab, with its divalent CD20-targeting structure, shown a higher affinity for its antigen. In a phase I study involving 177 NHL patients, 53% of patients treated with Glofitamab exhibited an ORR, with a CR rate of 36.8%. Among complete responders, 78% sustained their CR at 12 months during a median follow-up of 12.6 months 40. In recent years, numerous clinical trials have investigated dual-targeted CAR-T therapies in BCL, including various structural designs for dual targets and drug combination therapies, primarily with PD-1 inhibitors 19, 42, 66. Furthermore, innovative dual-target strategies have been explored for more effective treatments. For instance, CD19/CD20 dual-targeted CAR-T cells have been designed to treat both wild-type BCL and CD19-negative mutants, as well as B-NHL 67, 68; CD22/CD20 dual-target CAR-T cells demonstrated potent, durable, and dose-dependent activity *in vitro* and *in vivo* against primary B-NHL 69. Additionally, combining CD79b/CD3 bispecific antibodies (bsAbs) with CD19 CAR-T cells offers a promising clinical strategy 70. Other combinations, such as CD19/CD79a and CD3/CD20, are also being explored for the treatment of R/R BCL 43, 71.

#### Acute leukemia

Acute leukemia is classified into two main subtypes: acute lymphoblastic leukemia (ALL) and acute myeloid leukemia (AML). ALL is primarily characterized by either T-lineage or B-lineage involvement and is considered the most malignant in childhood 72. AML, on the other hand, is a rapidly progressive hematologic malignancy characterized by the clonal expansion and abnormal function of immature myeloid precursors. Chemotherapy and hematopoietic stem cell transplantation are two common treatment strategies for leukemia 73; however, the high rates of complications post-transplantation and a low 5-year survival rate seriously impact the overall therapeutic effect 74. Although CAR-T cell therapy has been utilized in the treatment of acute leukemia, challenges such as tumor antigen escape, severe CRS, and relapse after treatment are still observed in some patients 75, highlighting the need for novel therapeutic approaches.

Since the approval of Blinatumomab as the first bispecific antibody for B-ALL in 2017 49, numerous studies targeting dual antigens in leukemia have been initiated. One of the key challenges in CAR-T therapy for ALL is CD19-negative relapse, which remains a primary cause of treatment failure 76. To address this, a clinical study targeting CD19-negative NALM6 with CD19/CD22 dual-targeted CAR-T cells showed promising results, demonstrating long-lasting efficacy 46. In detail, the combination of CD19 and a novel CD22 CAR exhibited effective cytotoxicity even at low antigen densities. Twelve patients with advanced B-ALL were enrolled, and of these, 10 cases (83%) achieved measurable residual disease (MRD)-negative complete remission two months post-infusion. Furthermore, with a median follow-up of 8.7 months, none of the patients relapsed due to antigen-negative escape. The overall survival and event-free survival rates at 6 and 12 months were 75% and 60%, respectively. To date, a series of preclinical studies and clinical trials involving dual-targeting CD19/CD22 are actively being conducted in leukemia 47, 48, 77. C-type lectin-like molecule 1 (CLL-1) is a transmembrane glycoprotein, and CD123, another transmembrane glycoprotein, is part of the interleukin-3 (IL-3) receptor alpha chain. Previous studies have shown that leukemic stem cells (LSCs) play a critical role in leukemia onset and relapse. Both CD123 and CLL-1 are highly expressed on the surface of most leukemia cells, including LSCs, making them promising therapeutic targets for AML 78-80. For instance, Wang et al. 81 developed tandem CAR-T cells targeting CLL-1 and CD123 to assess their therapeutic potential in AML *in vitro*, and they found that these dual-targeting CAR-T cells exhibited robust killing effects and released a large number of cytokines, demonstrating a significant ability to kill single antigens and multi-target tumour cells. CD33, as a myeloid differentiation antigen, is also highly expressed on the blasts and LSCs in AML patients, while it is almost absent from normal hematopoietic stem cells 82. Clinical therapies targeting CD33 can effectively eliminate the transformed clone, they often fail to eradicate the precursor LSCs, which can lead to disease relapse 83. IL-10 has been shown to enhance the stemness of AML cells through various signaling pathways, and CAR-T cells targeting IL-10R have demonstrated cytotoxic effects against AML cells. A recent study developed anti-IL10R CAR-T cells that secrete CD33-targeting bsAbs, aimed at combating tumor heterogeneity and eradicating both LSCs and AML blasts. Moreover, these CAR-T cells, which deliver bsAbs directly to the tumor sites, can help overcome pharmacokinetic challenges and improve therapeutic efficacy 84. In addition, several other dual-target strategies have emerged in the past two years to address antigen escape in various forms of leukemias 85-89. Notable dual-target combinations include NKG2D/PD‑L1, IL-3 -zetakine /CD33, CD33/CD146, CD19/BAFF-R, and CD123/NKG2DLs. TCRs represent another promising approach for targeting tumor-derived neoantigens, as they can reduce the risk of off-tumor effects. TCR-based therapies have been considered highly specific alternatives for treating leukemia 90. For example, Teppert et al. 91 designed CAR'TCR-T cells co-expressing dNPM1-TCR and CD33-CAR for AML treatment. This strategy significantly enhanced anti-tumor cytotoxicity, demonstrating the potential of co-expressing both CAR and transgenic TCRs within a single T cell. Furthermore, ongoing studies are investigating the antibody-TCR dual-target approach, combining Wilms tumor 1 protein (WT1) and CD33, which is showing promise in early trials 92. These developments suggest that integrating TCRs into CAR-T cell therapies may open up new therapeutic avenues for AML and other malignancies.

### Applications of dual-targeted CAR-T in solid tumors

#### Hepatocellular carcinoma

Primary liver cancer is a global health issue, ranking as the sixth most common cancer worldwide and the fourth leading cause of cancer-related mortality. Hepatocellular carcinoma (HCC), accounting for 75-80% of liver cancers, is the predominant histological type 110, 111. The etiology of HCC is primarily rooted in chronic hepatitis, hepatitis B virus (HBV)/hepatitis C virus (HCV)-induced cirrhosis, alcoholic cirrhosis, dietary aflatoxin exposure, non-alcoholic steatohepatitis, alpha-1-antitrypsin deficiency, and hemochromatosis 112. Current clinical treatment options for HCC include surgical intervention, CAR-T cell therapy, immune checkpoint inhibitors (ICIs), tyrosine kinase inhibitors (TKIs), and antibody therapies. However, HCC remains largely incurable due to tumor heterogeneity and metastasis 113, 114. Recent research on immunotherapies employing dual-targeting strategies offers a promising new approach to overcoming the challenges of current treatments. Glypican 3 (GPC3), a tumor-associated antigen, is highly expressed in over 70% of HCC cases, but is strictly suppressed in normal liver tissue 115, 116. Fibroblast activation protein (FAP) is a type-II transmembrane serine protease secreted from cancer-associated fibroblasts (CAFs). During liver carcinogenesis, FAP promotes fibrosis in response to early liver injury, then facilitates tumor cell proliferation, and contributes to immune suppression 117-119. While CAR-T cells targeting GPC3 alone have shown some therapeutic efficacy, their clinical use is limited by several challenges 120. In response, bispecific CAR-T cells incorporating tandem scFvs targeting both FAP and GPC3 scFv have been developed to recognize and eliminate tumor cells expressing either or both antigens 39. Both *in vitro* and *in vivo* studies have shown that these dual-targeted CAR-T cells significantly enhance therapeutic efficacy against HCC, particularly in tumors with high expression of GPC3 or FAP. These cells suppress tumor growth and prolong survival in tumor-bearing mice, highlighting their potential to prevent antigen escape and combat heterogeneous HCC. ICIs, such as anti PD-1/PD-L1 monoclonal antibodies, have shown durable responses in a subset of HCC patients, particularly those with high PD-L1 expression or tumor mutational burden, however, the response rates in HCC are generally low (10-20%), and acquired resistance is common due to compensatory upregulation of alternative immune checkpoints (e.g., CTLA-4, TIM-3) 121, 122. To address this, a recent study developed a dual-targeting CAR-T cell (GPC3/PD-1) that recognizes GPC3 combining antigen-specific targeting PD-1 to block immune checkpoint, potentially overcoming ICI resistance by directly targeting tumor cells while modulating the TME. This strategy demonstrated greater resistance to PD-1/PD-L1 pathway inhibition, characterized by reduced inhibitory receptor expression and a less differentiated phenotype, resulting in more potent anti-tumor activity compared to single-target CAR-T cells 93. Epidermal Growth Factor Receptor (EGFR), a receptor tyrosine kinase (RTK) of the ErbB family, is highly expressed in human HCC and is associated with more aggressive tumor growth. It is also expressed at lower levels in liver epithelial cells, 123, 124. Combining GPC3 with EGFR, third-generation GPC3-EGFR CAR-T cells have been designed, showing enhanced proliferation and cytotoxicity while minimizing non-tumor toxicity 95. Furthermore, Chen et al. 97 constructed CAR-T cells carrying complementary CARs against GPC3 and ASGR1 (a liver tissue-specific protein). These dual-targeted CAR-T cells reduced the risk of on-target, off-tumor toxicity while maintaining anti-tumor activity in dual-positive HCC. In addition to these approaches, other dual-targeting strategies for HCC treatment are being explored to address antigen escape and the challenges posed by tumor microenvironment, such as targeting c-Met/PD-L1 and CD133/GPC3 94, 96.

#### Ovarian cancer

Ovarian cancer (OC) is the most lethal gynecological malignancy, with a poor clinical prognosis due to its high metastatic potential, drug resistance, and the lack of early detection and screening technologies 125. In addition to standard treatments such as surgery, radiotherapy, chemotherapy, and targeted therapies, cell immunotherapy, particularly CAR-T cell therapy, has emerged as an effective approach for cancer treatment 126, 127. MUC16 (also known as CA125) is a cell surface mucin that is highly expressed in epithelial ovarian tumors, making it a prominent marker in OC development 128. Recent findings have shown that MUC16 can suppress antitumor activity of immune cells, facilitating immune evasion by tumor cells 129, 130. Furthermore, Wilms tumor 1 (WT1), an intracellular transcription factor, is commonly overexpressed in various hematological and solid cancers including OC 131. To overcome the challenge of low MUC16 expression on ovarian cancer cells, MUC16-specific CAR-T cells were engineered to secrete a bispecific T cell engager that targets WT1, enabling the CAR-T cells to kill ovarian cancer cells via an orthogonal mechanism. This dual-target approach addresses tumor heterogeneity and enhances therapeutic efficacy by utilizing two distinct killing mechanisms 98. This study is the first to demonstrate the combination of targeting both intracellular and extracellular antigens, reducing the risk of antigen escape and improving overall treatment effectiveness. NKG2D is an activating receptor on NK cells that plays a crucial role in mediating immune cell activation and the destruction of target cells 132. Previous research has shown that over 80% of human ovarian cancer ascites samples express NKG2D ligands on their surface, and various NKG2D ligands are also found in human ovarian cancer cell lines 133, 134. PD-1 is another key target in cancer immunotherapy. Given the widespread expression of NKG2D and PD-1 ligands in various human cancers, these factors have been considered as promising targets for cancer treatment. Jiang et al. 100 combined a first-generation CAR targeting NKG2D ligands with a CAR targeting PD-1 ligands, generating a novel dual CAR that demonstrated broad clinical potential in precision cancer immunotherapy. Moreover, a tandem PD1-antiMUC16 dual CAR-T cell therapy has been developed. Data from preclinical studies showed that these dual CAR-T cells significantly enhanced the cytotoxicity against ovarian cancer OVCAR-3 cells and extended the survival time of tumor-bearing mice. This dual-targeted CAR-T approach exhibited more potent antitumor activity *in vivo* compared to single CAR-T cell therapies 99. Other dual-targeting strategies, such as targeting TAG-72/CD47 101, are also under investigation. These dual-target approaches have the potential to effectively prevent immune escape in OC and address challenges posed the tumor microenvironment, improving upon the limitations of single-target CAT therapies.

#### Glioblastoma multiforme

Glioblastoma multiforme (GBM) is the most common and aggressive form of primary malignant brain tumor, representing the highest grade of astrocytoma 135. GBM is characterized by its high invasiveness and resistance to almost all therapeutic interventions, including the combination of chemotherapy and radiotherapy following surgical resection. The challenges in treating GBM primarily arise from the drug resistance of malignant glioblastoma cells, as well as the complex distribution of inter- and intra-tumoral heterogeneity. As a result, the 5-year overall survival rate remains below 10% after treatment 136, 137. While current research on single-target antigens has shown promising therapeutic effects, complete and durable responses are rare, making it difficult to achieve robust efficacy 138. Therefore, developing dual-targeting strategies has become essential to address these challenges.

The epidermal growth factor receptor variant III (EGFRvIII) and the interleukin-13 receptor alpha 2 (IL-13Rα2) are specially expressed on the surface of GBM cells 139, yet are either completely absent or minimally expressed in normal somatic tissues. Given that EGFRvIII and IL-13Rα2 are co-expressed in the same tumor cells, the intracellular tandem specificity between these two molecules may confer a growth advantage to the tumor, making them an ideal combination for simultaneous targeting 140. A recent study developed a novel bispecific tandem CAR-T (TanCART) cell capable of targeting both EGFRvIII and IL-13Rα2. The results demonstrated that TanCAR-T cells exhibited enhanced activity and potent cytotoxicity, achieving complete and durable tumor responses in a heterogeneous GBM mouse model 20. This study highlights the effectiveness of TanCART in targeting heterogeneous brain tumors and provides further evidence supporting the development of multispecific CAR-T cell therapies for GBM. Transforming growth factor-beta (TGF-β) is overexpressed in gliomas and plays a critical role in maintaining the GBM tumor microenvironment by promoting the tumorigenicity of glioma-initiating stem cells, as well as tumor cell proliferation, invasiveness, and immune evasion 141, 142. A recent study designed a single-chain bispecific CAR targeting IL-13Rα2 and TGF-β. This CAR programs tumor-specific T cells to convert TGF-β from an immunosuppressive agent into an immunostimulatory one, thereby reshaping the immunosuppressive TME and enhancing antitumor responses in GBM 102. Treatment with IL-13Rα2/TGF-β CAR-T cells in human and mouse GBM models has demonstrated increased T cell infiltration, reduced levels of suppressive myeloid cells in the tumor-bearing brain, and improved survival rates in patient-derived GBM xenografts and syngeneic mouse models. This study offers a promising and novel therapeutic approach for the clinical translation of bispecific IL13Rα2/TGF-β CAR-T cells to overcome the immunosuppressive TME in GBM. Additionally, Saleh et al. generated 103 RevCAR T cells targeting both EGFR and disialoganglioside (GD2), which marked the first successful application of RevCAR T cells in a dual-targeting approach to efficiently, specifically, and programmably eliminate GBM cells both *in vitro* and *in vivo*.

#### Other solid tumors

In addition to extensive research on solid tumors, there has been a growing focus in recent years on dual-targeting approaches for various types of solid tumors. For instance, CD87×CD3 BiTE antibodies and CD87/IL-12 CAR-T cells have been designed to target non-functioning pituitary adenomas (NFPA) 104. The CD87×CD3 BiTE antibody effectively reduces tumor cell proliferation, demonstrating significant lytic activity both in tumor cells and in preclinical models. Furthermore, CD87/IL-12 CAR-T cells exhibited enhanced antitumor activity, inducing tumor regression more effectively than CD87 single-target CAR-T cells in both *in vivo* studies and three-dimensional co-culture models. Moreover, bispecific therapies targeting TGF-β and PD-L1 in various tumors, including lung, breast, and colorectal cancers, have been shown to significantly enhance T cell activation and cytotoxic responses 105, 143, 144. Dual-functional CAR-T cells targeting c-Met and PD-1, with PD-1 blocking capability, significantly boosted the cytotoxicity of CAR-T cells, demonstrating a stronger ability to inhibit tumor growth and prolong the survival of tumor-bearing mice in gastric cancer models 108. Dual CAR-T cells targeting CD276/FGFR4 effectively killed of rhabdomyosarcoma (RMS) cells *in vitro* and eradicated *in situ* RMS in preclinical models 106. Increasing numbers of Phase I clinical trials are investigating the safety and efficacy of bispecific antibodies targeting PD-1 and LAG-3 across various cancers, including epithelial ovarian cancer (EOC), triple-negative breast cancer (TNBC), non-small cell lung cancer (NSCLC), small cell lung cancer (SCLC), cervical cancer, and cholangiocarcinoma 145. Moreover, patients with refractory primary central nervous system lymphoma (PCNSL) have achieved long-term complete remission following treatment with CD19/CD22 dual-targeting CAR-T cells in combination with PD-1 and BTK inhibitors 146. CAR-T cells targeting CD30 and carcinoembryonic antigen (CEA) have shown improved redirected immune responses against colorectal cancer 21. Furthermore, CAR-T cells targeting both CEA and mesothelin (MSLN) exhibit potent antitumor activity in pancreatic cancer, significantly inhibiting tumor cell growth without affecting normal tissues 109. These examples highlight the increasing momentum of dual-targeting studies across a wide range of solid tumors. In summary, dual-targeting CAR-T cells demonstrate enhanced persistence within tumor tissues, reduced expression of inhibitory receptors, and a less differentiated phenotype, thereby achieving a more potent and sustained antitumor effect.

## Discussion and Prospects

Despite the promising preclinical and clinical outcomes of dual CAR-T cell therapy for both hematological and solid tumors, several challenges remain before its widespread clinical application. This section discusses the current limitations of dual CAR-T cell therapy, potential strategies to address these challenges, and future directions for development.

First, the complexity of designing the dual CAR structure of CAR-T cells poses a major challenge. Developing appropriate vector systems is essential for enhancing the functionality of CAR-T cells 147. The rational design of two CAR domains is critical to avoid internal competition between the domains and to ensure efficient dual-specific recognition. In addition, the high manufacturing costs and complex production processes of dual CAR-T cells may limit their accessibility, particularly in low- and middle-income countries 16, 148. Second, the safety profile of dual-targeted CAR-T cells is a critical consideration for the clinical translation. In a clinical trial for non-Hodgkin lymphoma, tandem CD19/CD20 CAR-T cells achieved a median PFS of 23.9 months, significantly longer than single-target CAR-T therapies (8.9 months), with no increased incidence of CRS or neurotoxicity 149. Although dual-targeted CAR-T cells can mitigate on-target/off-tumor toxicity by targeting tumor-specific antigen pairs, they face unique challenges including TME-mediated exhaustion and and cytokine storm risk 11. Emerging strategies address these risks through engineering innovations, such as logic-gated activation, metabolic reprogramming, and epigenetic modulation 150. For instance, AND-gated EGFRvIII/IL13Rα2) significantly reduced uncontrolled cytokine release 151. IL-6 receptor blockade (tocilizumab) combined with short-course corticosteroids remains first-line therapy, resolving grade 3-4 CRS in 80% of cases 152. Moreover, decitabine-primed tandem CD19/CD22 dual-targeted CAR-T therapy maintained complete remission for a 35-month follow-up period without neurotoxicity 146. Also, the FDA emphasizes key insights and recommendations for optimizing safety, including safety switches, dose optimization, and biomarker monitoring, as highlighted in the FDA's guidance document *Considerations for the Development of Chimeric Antigen Receptor (CAR) T Cell Products*. Furthermore, the specificity and efficacy of dual-targeted CAR-T cells across diverse tumor types require validation through large-scale clinical trials with extended follow-up periods 153. To enhance therapeutic outcomes, strategic approaches can be prioritized, such as optimizing co-stimulatory signals in CAR-T cells (e.g., combining CD28 and 4-1BB domains), improving the tumor microenvironment (e.g., immunosuppressive cell depletion, physical barrier disruption, hypoxia and metabolic stress alleviation), and exploring novel dual CAR-T cell designs (e.g., logic-gated CARs) to address antigen heterogeneity and enhance persistence 11, 28, 154, 155.

In recent years, advancements in gene-editing technologies (e.g., CRISPR-Cas9) and a deeper understanding of the tumor immune microenvironment have paved the way for more personalized and precise treatments using dual CAR-T cells, enabling enhanced targeting of heterogeneous tumors and overcoming immune evasion mechanisms 156, 157. Moreover, combination therapies incorporating immune checkpoint inhibitors, oncolytic viruses, or other immunomodulatory agents may further enhance the therapeutic efficacy of dual-targeted CAR-T cells, offering new hope for cancer patients. As clinical research continues to progress, dual-targeted CAR-T cell therapy is expected to become a significant modality in cancer treatment in the near future.

## Supplementary Material

Supplementary table.

## Figures and Tables

**Table 1:** The outcomes of clinical trials of dual-targeted CAR-T therapy in hematological malignancies.

Disease	dual-target	ClinicalTrials. number	Phase	Number of subjects	Complete remission rate (CR)	Overall survival rate (OS)	Ref.
B-NHL	CD19/CD22	ChiCTR1800015575	Phase I	16	62.5%	77.3%	19
B-NH	CD3/CD20	NCT03075696	Phase I	177	36.8%	/	40
BCL	CD19/CD20	NCT03233854	Phase I	21	/	77%	41
BCL	CD19/CD22	NCT03289455	Completed	52	17%	/	9
BCL	CD19/CD22	ChiCTR2100052247	Completed	24	/	90%	42
BCL	CD3/CD20	NCT03625037	Phase I/II	157	38.9%	/	43
MM	BCMA/CD19	ChiCTR2000033567	Phase I/II	50	/	/	44
MM	BCMA/CD19	NCT02546167	Phase I	30	/	/	45
ALL	CD19/CD22	NCT02443831	Phase I	12	/	75%	46
ALL	CD19/CD22	NCT04227015	Phase I	6	83.3%	/	47
ALL	CD19/CD22	NCT03289455	Phase I	15	86%	60%	48
ALL	CD19/CD3	NCT02013167	Phase III	271	12%	/	49

Notes: B-NHL: B-cell Non-Hodgkin Lymphoma; BCL: B‑Cell Lymphoma; BCMA: B-Cell Maturation Antigen; MM: Multiple Myeloma; ALL:Acute Lymphoblastic Leukemia.

**Table 2:** The outcomes and efficacy of dual-targeted CAR-T therapy in tumor-bearing mouse models.

Disease	Dual-target	Mouse model	Groups of mice	Single-CAR mouse survival rate	Double-CAR mouse survival rate	Tumor volume changes in single CAR mouse	Tumor volume changes in double CAR mouse	Ref.
HCC	GPC3/FAP	HepG2 CDX	5	40%(day50)	60%(day50)	Increase	Increase slowly	39
HCC	GPC3/PD-1	TX	4	80%(day40)	100%(day40)	Increase	Decrease	93
HCC	GPC3/ CD133	Huh-7	5	0(day75)	80%(day75)	No Change	Decrease	94
HCC	GPC3/EGFR	Huh-7-luc	5	10%(day52)	50%(day52)	Decrease	Significantly decrease	95
HCC	c-Met/PD-L1	HepG2-fLuc	4	40%(day50)	80%(day50)	Decrease	Significantly decrease	96
HCC	GPC3/ASGR1	Huh-7/MHCC-97L	4	/	/	No Change	Significantly decrease	97
EOC	Muc16/ WT1	SKOV3/A2+/GFP+	4	20%(day60)	50%(day60)	Decrease	Significantly decrease	98
EOC	MUC16/ PDL-1	OVCAR3-MUC16GFP-PDL1-luc	4	48%(day40)	100%(day40)	No change	Significantly decrease	99
OC/CC	NKG2D / PDL-1	HCT116-Luc/ SKOV3-Luc	4	0%(day60)	100%(day60)	No change	Significantly decrease	100
OC	TAG-72 /CD47	TAG-72high OVCAR-3/ TAG-72low MESOV	4	/	/	No change	Decrease	101
GBM	IL-13Rα2/TGF-β	GS001-NSG/C57BL6	5	20%(day100)	75%(day100)	Decrease	Significantly decrease	102
GBM	EGFRvIII /IL-13Rα2	U87MG-NSG	4	68%(day50)	100%(day50)	Decrease	Significantly decrease	20
GBM	EGFR/GD2	U251 Luc-NXG	5	/	/	Decrease	Significantly decrease	103
iNFPAs	CD87/CD3; CD87/IL-12	iNFPA PDXs	5	20%(day55)	90%(day55)	Decrease	Significantly decrease	104
Breast/lung/ colorectal cancer	TGF-β/ PDL-1	HCT116- Hsd	5	0%(day53)	50%(day53)	No change	Significantly decrease	105
RMS	CD276/FGFR4	iRFP720+fLuc+ RMS	5	60%(day40)	100%(day40)	Decrease	Significantly decrease	106
Solid tumors	CD19/BCMA	HEp-2 / PC-3 /ECA109-NCG	4	/	/	Decrease	Significantly decrease	107
Solid tumors	c-Met/PD-1	MKN45/A549-NOD/SCID	5	40%(day40)	80%(day40)	Decrease	Significantly decrease	108
Pancreatic cancer	CEA/ MSLN	AsPC-1/HT29/U87/PANC-1	6	/	/	Decrease	Significantly decrease	109

Notes: HCC, Hepatocellular Carcinoma; EOC, Epithelial Ovarian Cancer; OC, Ovarian Cancer; CC, Colon Cancer; GBM, Glioblastoma Multiforme; iNFPAs: invasive nonfunctioning pituitary adenomas; RMS, Rhabdomyosarcoma.
