# Supplementary material for: From molecular design to clinical translation: dual-targeted CAR-T strategies in cancer immunotherapy
Source: Int J Biol Sci. 2025 Mar 31;21(6):2676–91. doi: 10.7150/ijbs.108036 (PMC12035882; doi:10.7150/ijbs.108036)
Supplement: Supplementary file 1 — Supplementary table. [file ijbsv21p2676s1.pdf]

Table S1 Comparison of structural differences dual-targeted CARs (Tandem CAR, Parallel CAR, and synNotch CAR).

Characteristics	Tandem CAR	Parallel CAR	synNotch CAR
**Structure**	Single chain with two scFvs	Two independent CARs on one T cell	Primary CAR + synNotch receptor
**Target Recognition**	Simultaneous binding to two antigens	Independent binding to two antigens	Sequential binding (Antigen A → Antigen B)
**Signaling Pathway**	Integrated CD3ζ + co-stimulation	Separate CD3ζ for each CAR	synNotch → Primary CAR activation
**Activation Logic**	OR-gated (either antigen activates)	OR-gated (either antigen activates)	AND-gated (both antigens required)
**Potential Advantages**	Compact design, easy manufacturing	Flexible antigen pairing	High specificity, reduced off-target toxicity
**Limitations**	Potential steric hindrance	Higher manufacturing complexity	Delayed activation kinetics
